# Synthesis of Diarylpyrazoles Containing a Phenylsulphone or Carbonitrile Moiety and their Chalcones as Possible Anti-Inflammatory Agents

**DOI:** 10.3797/scipharm.1105-14

**Published:** 2011-07-03

**Authors:** Ekhlass Nassar, Hatem A. Abdel-Aziz, Hany S. Ibrahim, Ahmed M. Mansour

**Affiliations:** 1 Department of Chemistry, Faculty of Women for Arts, Science and Education, Ain Shams University, Cairo, Egypt; 2 Department of Pharmaceutical Chemistry, College of Pharmacy, King Saud University, P.O. Box 2457, Riyadh 11451, Saudi Arabia; 3 Department of Pharmaceutical Chemistry, Faculty of Pharmacy, Egyptian Russian University, Badr City, Cairo 11829, Egypt; 4 Department of Pharmacology & Toxicology, Faculty of Pharmacy, Al-Azhar University, Nasr City, Cairo 11884, Egypt

**Keywords:** Diarylpyrazoles, Sulphones, Chalcones, Anti-inflammatory activity, Molecular docking

## Abstract

A series of chalcone-based diarylpyrazoles containing a phenylsulphone or carbonitrile moiety was synthesized. Thus, 3-acetylpyrazoles **6a–c** and **10a–c** were used as useful substrates in facile synthesis of functional pyrazoles **7a–f** and **11a–f**, respectively. The anti-inflammatory activity and ulcerogenic effect were evaluated and some of the obtained products possessed a significant anti-inflammatory activity. 1-[1-(3-Methylphenyl)-5-phenyl-4-(phenylsulfonyl)-1*H*-pyrazol-3-yl]ethanone (**6b**) showed a high activity when compared with indomethacin as reference drug with lower gastrointestinal (GI) profile. Furthermore, molecular docking studies were performed in order to rationalize the obtained biological results.

## Introduction

Management of inflammatory disorders involves a stepwise approach to the use of therapeutic agents. Relieving of pain and reduction of inflammation are urgent goals to reduce the severity of symptoms. A generally accepted stepwise approach to treat the inflammation disorders includes physical therapy, non-steroidal anti-inflammatory drugs (NSAIDs), disease modifying anti-rheumatic drugs (DMARDs), corticosteroids and finally, immunosuppressive agents [1, 2]. However, NSAIDs remain among the most widely prescribed drugs worldwide; they have been generally considered as inhibitors of cyclooxygenases (COXs) [3, 4]. Most of NSAIDs act by reducing prostaglandin biosynthesis through the inhibition of the COX reaction [5]. Moreover, data have been accumulating through the years suggesting that NSAIDs also probably act on other targets to counteract pain. In spite of their beneficial action, their activity is associated with deleterious side effects, and continuous administration of these drugs leads to nephrotoxicity and gastric ulcerations. The therapeutic anti-inflammatory action of NSAIDs is produced by inhibition of COX-2, while the unwanted side effects arise from the inhibition of COX-1 activity. It is estimated that 25% of patients using NSAIDs experience some kind of side effect and about 5% develop serious health consequence (massive GI bleed, acute renal failure, etc.). Consequently, extensive research has been directed towards improving their pharmacological profile [6].

**Fig. 1.**
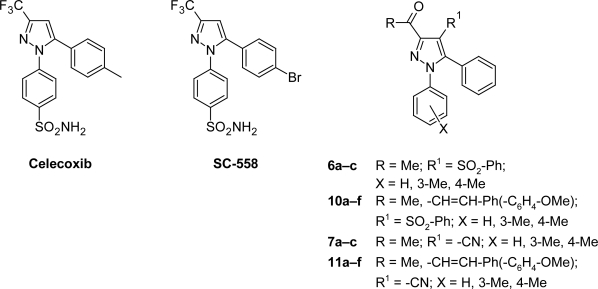
Structure of Celecoxib, SC-558, **6a–c**, **10a–f**, **7a–c** and **11a–f**.

Recently, a novel class of selective COX-2 inhibitors has been discovered. Among this class, celecoxib (Figure 1), the potent and gastrointestinal (GI) safe anti-inflammatory agent. It is considered a typical model of sulphonamide-based diarylpyrazole template that is known to selectively inhibit COX-2 [7–15]. Furthermore, several arylpyrazoles are available as anti-inflammatory drugs in market such as valdecoxib [16], rofecoxib [17], etoricoxib [18, 19]. SC-558, diarylpyrazole derivative (Figure 1), was found to be a marvelous inhibitor with 1900-fold selectivity for COX-2 over COX-1. In addition, both SC-558 and celecoxib were published, co-crystallized with the active site of COX-2, with many investigations about their mechanism of action and selectivity to COX-2 [20–22].

In view of the above facts and in continuation of our interest in the synthesis of bioactive heterocycles, especially pyrazole derivatives [23–32], we report here some di/triaryl-pyrazoles having phenylsulphone or carbonitrile moiety (Figure 1). The anti-inflammatory activity of the synthesized compounds was evaluated. Moreover, molecular docking studies of active compounds were carried out to rationalize their activity.

## Results and Discussion

### Chemistry

Cyclization reaction of hydrazonoyl chlorides **1a–c** with 1-phenyl-2-(phenylsulfonyl)-ethanone (**2**) in ethanolic sodium ethoxide solution at room temperature furnished 1-(5-phenyl-4-(phenylsulfonyl)-1-aryl-1*H*-pyrazol-3-yl)ethanones (**6a–c**), respectively (Scheme 1). The latter pyrazoles were reacted with benzaldehyde or 4-anisaldehyde in ethanol containing 10% aqueous sodium hydroxide at room temperature to afford chalcones **7a–f**. The IR spectra of the latter chalcones showed, in each case, the appearance of absorption band in the region 1640–1676 cm^−1^ corresponding to the carbonyl function. Their ^1^H NMR spectra showed the disappearance of COCH_3_ protons signal and displayed the signals of olefinic protons in the aromatic region.

Similarly, the reaction of 3-acetyl-(1-aryl)-5-phenyl-1*H*-pyrazole-4-carbonitriles (**10a–c**) with benzaldehyde or 4-anisaldehyde, under the same reaction conditions for synthesis of **7a–f**, afforded the corresponding chalcones **11a–f**, respectively (Scheme 2). The structure of the latter chalcones was confirmed under the basis of their spectral data. For example, their IR spectra revealed the appearance of absorption band in the region 1662–1668 cm^−1^ due to the carbonyl group in addition to the appearance of sharp absorption peak of carbonitrile function around 2235 cm^−1^ and their mass spectra showed, in each case, a peak corresponding to their molecular ion.

## Pharmacology

### Anti-inflammatory activity

The anti-inflammatory effect of tested compounds, meloxicam and indomethacin, on carrageenan-induced edema at 3 and 6h, is depicted in Table 1. Percent edema inhibition (Table 1) was calculated in regard to control group and the potency (%) was calculated respect to the indomethacin response. After 3h, the tested compounds **6a–c**, **7b**, **7d**, **10a** and **11b** showed a reasonable decrease in the edema size ranging between 30.12% for compound **6c** and 105.51% for the most active compound **6b**. On the other hand, compounds **11c–f** showed a weak activity in reduction of edema size after the same time (< 27.34 % for **11e**) as shown in Table 1. After 6h, **6b** was still the most potent anti-inflammatory compound in minimizing the inflammation size with 99.82% activity. However, compounds **7a**, **7c**, **7e**, **7f**, **10b**, **10c** and **11a** showed no inhibitory effect (Table 1).

From the structure activity relationship (SAR) viewpoint, the anti-inflammatory activity of phenylsulfonylpyrazoles such as **6a**, **6c**, **7b** and **7d** were found to be higher than that of carbonitrile pyrazoles except **10a** which achieved a greater activity. Additionally, the introducing of chalcone moiety to pyrazole ring was found to be variable in effectiveness of activity. However, these results imply that the phenyl sulphone moiety and carbonyl group attached to the pyrazole system is an essential pharmacophore responsible for activity. Furthermore, changing the position of 4-methyl substituent of *N*-phenyl ring in **6c** to position 3 in **6b** increased the activity. On the other hand, the phenylsulphonylpyrazoles that contains 4-methoxybenzylidine group, **7b** and **7d**, showed a higher activity while that with benzylidine group, **7a** and **7c**, have no activity. Taken together, the results of **6a–c** and **7a–f** provide evidence that the structural features of arylidine moieties and changes in the *N*-aryl group significantly affects their anti-inflammatory activity.

### Ulcerogenic effects

The most active compounds **6b**, **7b**, **7d** and **10a** were selected for the ulcerogenic study as compared to the reference drugs meloxicam and indomethacin [33]. Compound **6b** showed lower ulceration (1.5) respect to meloxicam (1.67) or to indomethacin (3.17) while compounds **7b**, and **7d** recorded ulcer index higher than meloxicam but still lower than indomethacin; compound **10a** showed the highest ulcer index (2.83) (Table 2).

## Molecular docking

The docking runs were conducted using Molsoft ICM-pro software to rationalize the obtained biological results. Besides, the molecular docking studies helped in understanding the various interactions between the ligand and enzyme active sites. An automated docking study was carried out using the crystal structure of COX-2 (pdb ID: 1CX2) in which SC-558 co-crystallized as a ligand [20]. Both enzymes were submitted to regularization process to fit the protein model with the ideal covalent geometry of residues to the atom positions of a target PDB structure [34]. The regularized protein was used in determination of the important amino acids in ligand binding pocket (LBP) of COX-2 enzyme.

**Fig. 2.**
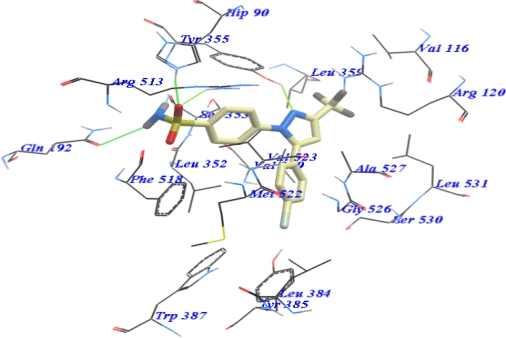
Binding mode of SC-558 with COX-2 active site. The hydrogen bonds with amino acid residues His90, Tyr355, Arg513, Gln192 & Ser353 represented by green lines.

Re-docking of the SC-558 was done to investigate its interaction with active site of COX-2 enzyme. Binding mode of SC-558 showed the binding of bromophenyl ring in a hydrophobic cavity by Gly526, Leu384, Tyr385, Trp387, Met522, Phe518, Val523 and Ser530 interaction (Figure 2). The trifluoromethyl group was bounded in an adjacent pocket by Val116, Tyr355, Leu359 and Leu531. The sulphonamide group extends into a relatively polar region and interacts with His90, Arg513, Gln192, Leu352 and Ser353 [20, 35]. Interactive docking using Mol table ligand was carried out for all the conformers of all of the tested compounds with remarked biological activity to the selected active site of COX-2. Each docked compound was assigned by a score according to its fitting to LBP and its binding mode with different amino acid residues that have a role in its biological activity (Table 3).

**Fig. 3.**
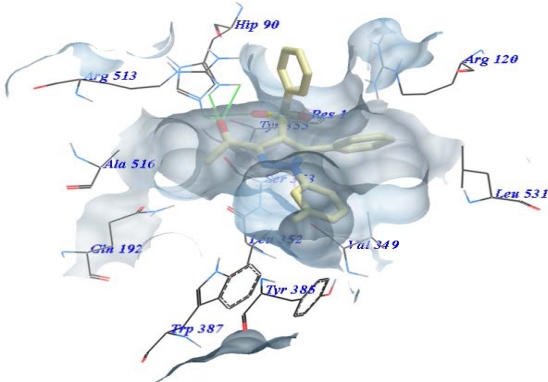
Docking of compound 6b inside the LBP of COX-2 showing the hydrogen bonds (green lines) and the stacks of aryl rings.

The interaction of 6b with COX-2 active site appeared that, sulphone function and carbonyl group were formed a hydrogen bonds with Tyr355, His90 and Arg513 (Figure 3). In addition, the acetyl group was imbedded in the hydrophobic pocket of Phe518, Ala516, Ser353 and Leu352. In addition, the *m-*methyl substituent in 6b was surrounded by a pocket of Gly526, Tyr385 and Trp387.

**Fig. 4.**
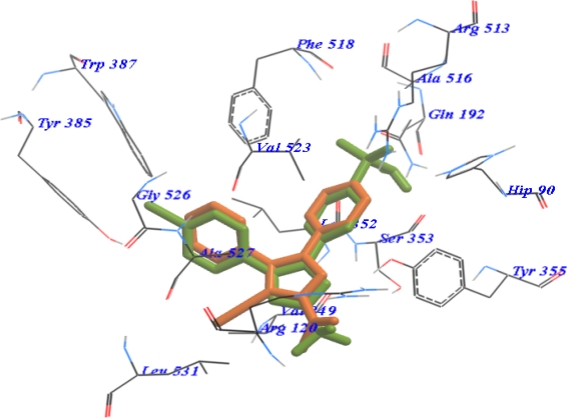
Overlay of SC-558 (green) and 10a (orange) inside the active site of COX-2.

Next, the binding mode of interaction of compound **10a** overlaid with SC-558 revealed the same alignment with SC-558. Nevertheless, compound **10a** showed a deficient of sulphonamide binding mode (Figure 4). Compounds **7b** and **7d** gave good binding scores and binding modes with different amino acids in the receptor of COX-2 respect to their biological activity.

## Conclusion

In conclusion, we described a facile synthesis of poly-functionally pyrazoles. Some of synthesized pyrazoles showed a significant anti-inflammatory activity. 1-(5-Phenyl-4-(phenylsulfonyl)-1-(3-tolyl)-1*H*-pyrazol-3-yl)ethanone (**6b**) possessed a high activity, when compared with reference drugs, with lower gastrointestinal (GI) profiles while compounds **7b**, **7d** and **10a** have a moderate anti-inflammatory activity. Molecular docking study gave us good information about the interaction mode of sulfone-based pyrazoles with COX-2 active site. They have the ability to make hydrogen bonds with amino acid residues Tyr355, His90 and Arg513 in COX-2 active site.

## Experimental

### Chemistry

Melting points (°C, uncorrected) were determined using a Gallenkamp melting point apparatus. Elemental analytical data were obtained from the microanalytical unit, Cairo University, Giza, Egypt. The IR spectra (KBr) were recorded on a PerkinElmer FT/IR spectrometer. At 400 MHz, the NMR spectra were recorded on a Jeol spectrometer using tetramethylsilane as an internal standard. ^1^H and ^13^C spectra were run at 400 and 100 MHz, respectively. Splitting patterns are designated as follows: s, singlet; d, doublet; t, triplet; m, multiplet. Chemical shift (δ) values are given in parts per million and coupling constants (*J*) in Hertz. The mass spectra were performed using a Varian MAT CH-5 spectrometer (70 eV).

#### 1-(5-Phenyl-4-(phenylsulfonyl)-1-aryl-1*H*-pyrazol-3-yl)ethanone (**6a–c**)

1-Phenyl-2-(phenylsulfonyl)ethanone (**2**) (2.6 g, 10 mmol) was added to a stirred ethanolic sodium ethoxide solution [prepared from sodium metal (0.23 g, 10 mmol) and 50 mL of absolute ethanol]. After stirring for 20 min, the appropriate 2-oxo-*N*'-arylpropane-hydrazonoyl chloride (**1a–c**) (10 mmol) was added and the reaction mixture was left to stir at room temperature for 12 h. Then added to cold water, the solid product was collected by filtration, washed with water and dried. Recrystallization from ethanol afforded pyrazoles **6a–c**. Synthesis of pyrazoles **6a** and **6c**, using ultrasonic, was reported earlier [36].

#### 1-[1-(3-Methylphenyl)-5-phenyl-4-(phenylsulfonyl)-1*H*-pyrazol-3-yl]ethanone (**6b**)

Pale yellow crystals, 74% yield; mp 166–168 °C; IR (KBr) ν_max_/cm^−1^ 1708 (C=O), 1610 (C=N); ^1^H NMR (DMSO-*d_6_*) δ 2.24 (s, 3H, *m*-CH_3_), 2.56 (s, 3H, -COCH_3_), 7.09–7.42 (m, 9H, ArH), 7.57–7.69 (m, 3H, ArH), 7.89 (d, 2H, *J* = 7.92 Hz, ArH); ^13^C NMR *δ* 21.21 (*m*-CH_3_), 28.73 (-COCH_3_), 127.44, 127.92, 128.36, 129.18, 129.43, 130.25, 130.46, 131.19, 133.83, 138.43, 139.25, 142.49, 148.37, 192.70 (-COCH_3_); MS *m/z* (*%*) 416 (M^+^, 1.93), 207 (100.0). Anal. Calcd for C_24_H_20_N_2_O_3_S (416.49): C, 69.21; H, 4.84; N, 6.73; S, 7.70. Found: C, 69.42; H, 4.93; N, 6.59; S, 7.53.

#### Synthesis of chalcones **7a–f**

To a stirred solution of pyrazole **6a–c** (10 mmol) and the appropriate aldehyde (10 mmol) in ethanol (30 mL), 10% aqueous sodium hydroxide (5 mL) was added portion-wise at room temperature for 10 min, the reaction mixture was further stirred for 12 h. The resulting solid was filtered off, washed with water, dried and crystallized from EtOH/DMF to afford chalcones **7a–f**.

#### (2*E*)-1-(1,5-Diphenyl-4-(phenylsulfonyl)-1*H*-pyrazol-3-yl)-3-phenylprop-2-en-1-one (**7a**)

White powder, 79% yield; mp 186–188 °C; IR (KBr) ν_max_/cm^−1^ 1646 (C=O), 1607 (C=N); ^1^H NMR (DMSO-*d_6_*) δ 7.38–7.90 (m, 22H, ArH); ^13^C NMR *δ* 121.89, 124.82, 127.00, 127.70, 127.91, 128.47, 129.53, 129.65, 129.79, 130.37, 131.22, 131,74, 133.92, 134.92, 138.59, 142.52, 146.80, 148.95, 185.21 (-C=O); MS *m/z* (*%*) 490 (M^+^, 0.95), 207 (100). Anal. Calcd for C_30_H_22_N_2_O_3_S (490.57): C, 73.45; H, 4.52; N, 5.71; S, 6.54. Found: C, 73.31; H, 4.57; N, 5.84; S, 6.42.

#### (2*E*)-1-(1,5-Diphenyl-4-(phenylsulfonyl)-1*H*-pyrazol-3-yl)-3-(4-methoxyphenyl)prop-2-en-1-one (**7b**)

White powder, 62% yield; mp 176–178 °C; IR (KBr) ν_max_/cm^−1^ 1676 (C=O), 1589 (C=N); ^1^H NMR (DMSO-*d_6_*) δ 3.82 (s, 3H, -OCH_3_), 7.01–7.04 (d, 2H, *J* = 8.8 Hz, ArH), 7.36–7.44 (m, 11H, ArH), 7.57–7.89 (m, 8H, ArH); ^13^C NMR *δ* 56.00 (-OCH_3_), 115.15, 121.78, 122.61, 126.96, 127.30, 127.73, 127.91, 128.47, 129.51, 129.74, 130.35, 131.22, 131.54, 133.88, 138.60, 142.58, 146.93, 147.58, 149.28, 185.31 (-C=O); MS *m/z* (*%*) 521 (M^+^+1, 0.70), 281 (36.82), 207 (100), 73 (41.93). Anal. Calcd for C_31_H_24_N_2_O_4_S (520.60): C, 71.52; H, 4.65; N, 5.38; S, 6.16. Found: C, 71.35; H, 4.48; N, 5.31; S, 6.30.

#### (2*E*)-1-[1-(3-Methylphenyl)-5-phenyl-4-(phenylsulfonyl)-1*H*-pyrazol-3-yl]-3-phenylprop-2-en-1-one (**7c**)

White powder, 65% yield; mp 110–112 °C; IR (KBr) ν_max_/cm^−1^ 1676 (C=O), 1610 (C=N); ^1^H NMR (DMSO-*d_6_*) δ 2.24 (s, 3H, *m*-CH_3_), 7.14–7.88 (m, 21H, ArH); ^13^C NMR *δ* 21.23 (*m*-CH_3_), 123.97, 124.81, 127.42, 127.72, 127.86, 128.44, 129.19, 129.51, 129.63, 130.38, 131.21, 131.72, 133.89, 134.67, 138.50, 139.24, 146.67, 147.69, 185.40 (-C=O); MS *m/z* (*%*) 504 (M^+^, 6.87), 207 (100). Anal. Calcd for C_31_H_24_N_2_O_3_S (504.60): C, 73.79; H, 4.79; N, 5.55; S, 6.35. Found: C, 73.85; H, 4.75; N, 5.47; S, 6.48.

#### (2*E*)-3-(4-Methoxyphenyl)-1-[1-(3-methylphenyl)-5-phenyl-4-(phenylsulfonyl)-1*H*-pyrazol-3-yl]prop-2-en-1-one (**7d**)

White powder, 63% yield; mp 169–171 °C; IR (KBr) ν_max_/cm^−1^ 1640 (C=O), 1597 (C=N); ^1^H NMR (DMSO-*d_6_*) δ 2.24 (s, 3H, *m*-CH_3_), 3.83 (s, 3H, -OCH_3_), 7.02–7.86 (m, 20H, ArH); ^13^C NMR *δ* 21.23 (*m*-CH_3_), 56.01 (-OCH_3_), 115.15, 121.70, 122.58, 123.94, 127.30, 127.39, 127.75, 127.85, 128.44, 129.18, 129.49, 130.33, 131.21, 131.55, 133.85, 138.51, 139.23, 142.59, 146.81, 147.50, 149.23, 185.30 (-C=O); MS *m/z* (*%*) 534 (M^+^, 2.56), 207 (100). Anal. Calcd for C_32_H_26_N_2_O_4_S (534.62): C, 71.89; H, 4.90; N, 5.24; S, 6.00. Found: C, 71.77; H, 4.91; N, 5.38; S, 5.93.

#### (2*E*)-1-[1-(4-Methylphenyl)-5-phenyl-4-(phenylsulfonyl)-1*H*-pyrazol-3-yl]-3-phenylprop-2-en-1-one (**7e**)

White fibers, 60% yield; mp 173–174 °C; IR (KBr) ν_max_/cm^−1^ 1642 (C=O), 1602 (C=N); ^1^H NMR (DMSO-*d_6_*) δ 2.26 (s, 3H, *p*-CH_3_), 7.17–7.89 (m, 21H, ArH); ^13^C NMR *δ* 21.15 (*p*-CH_3_), 121.80, 124.86, 126.74, 127.78, 127.89, 128.47, 129.51, 129.64, 129.93, 130.34, 131.20, 131.71, 133.88, 134.69, 136.18, 139.50, 142.56, 146.70, 147.69, 148.87, 185.22 (-C=O); MS *m/z* (*%*) 504 (M^+^, 0.57), 281 (39.65), 207 (100), 96 (24.96), 73 (45.89). Anal. Calcd for C_31_H_24_N_2_O_3_S (504.60): C, 73.79; H, 4.79; N, 5.55; S, 6.35. Found: C, 73.66; H, 4.91; N, 5.42; S, 6.38.

#### (2*E*)-3-(4-Methoxyphenyl)-1-[1-(4-methylphenyl)-5-phenyl-4-(phenylsulfonyl)-1*H*-pyrazol-3-yl]prop-2-en-1-one (**7f**)

White fibers, 61% yield; mp 178–180 °C; IR (KBr) ν_max_/cm^−1^ 1676 (C=O), 1593 (C=N); ^1^H NMR (DMSO-*d_6_*) δ (s, 3H, *p*-CH_3_), 3.82 (s, 3H, -OCH_3_), 7.01–7.88 (m, 20H, ArH); ^13^C NMR *δ* 21.23 (*p*-CH_3_), 56.00 (-OCH_3_), 115.15, 121.68, 122.66, 126.70, 127.32, 127.81, 127.88, 128.47, 129.50, 129.92, 130.31, 131.20131.51, 133.85, 136.20, 139.44, 142.62, 146.82, 147,49, 149.20, 185.32 (-C=O); MS *m/z* (*%*) 533 (M^+^, 0.50), 281 (17.83), 207 (100), 73 (29.26). Anal. Calcd for C_32_H_26_N_2_O_4_S (534.62): C, 71.89; H, 4.90; N, 5.24; S, 6.00. Found: C, 72.04; H, 5.06; N, 5.37; S, 5.88.

#### Synthesis of pyrazoles **10a–c**

Those compounds were synthesized using the method that described for synthesis of pyrazoles **6a–c** using benzoylacetonitrile **8** instead of sulphone **3**. pyrazoles **10a** and **10c** were described in literature [37].

#### 3-Acetyl-1-(3-methylphenyl)-5-phenyl-1*H*-pyrazole-4-carbonitrile (**10b**)

Pale yellow crystals, 70% yield; mp 160–162 °C; IR (KBr) ν_max_/cm^−1^ 2233 (C≡N); 1693 (C=O), 1610 (C=N); ^1^H NMR (DMSO-*d_6_*) δ 2.30 (s, 3H, *m*-CH_3_), 2.61 (s, 3H, -COCH_3_), 7.09–7.52 (m, 9H, ArH); ^13^C NMR *δ* 21.29 (*m*-CH_3_), 26.99 (-COCH_3_), 92.64, 123.58, 126.37, 126.83, 129.52, 129.62, 129.90, 130.81, 131.12, 138.39, 139.80, 151.01, 151.10, 192.30 (-COCH_3_); MS *m/z* (*%*) 301 (M^+^, 3.79), 207 (100). Anal. Calcd for C_19_H_15_N_3_O (301.34): C, 75.73; H, 5.02; N, 13.94. Found: C, 75.85; H, 4.89; N, 14.03.

#### Synthesis of chalcones **11a–f**

This reaction was carried out by the same procedure described in the synthesis of compounds **7a–f** using pyrazoles **10a–c** instead of **6a–c**.

#### 1,5-Diphenyl-3-[(2*E*)-3-phenylprop-2-enoyl]-1*H*-pyrazole-4-carbonitrile (**11a**)

White powder, 66% yield; mp 220–222 °C; IR (KBr) ν_max_/cm^−1^ 2237 (C≡N); 1664 (C=O), 1597 (C=N); ^1^H NMR (DMSO-*d_6_*) δ 7.42–7.98 (m, 17H, ArH); ^13^C NMR *δ* 92.60, 113.73, 121.65, 126.39, 126.64, 129.53, 129.56 129.69, 129.94, 130.00, 131.16, 131.80, 182.39 (-C=O); MS *m/z* (*%*) 375 (M^+^, 0.37), 281 (39.68), 207 (100), 91 (37.51), 73 (79.69). Anal. Calcd for C_25_H_17_N_3_O (375.42): C, 79.98; H, 4.56; N, 11.19. Found: C, 80.07; H, 4.58; N, 11.08.

#### 3-[(2*E*)-3-(4-Methoxyphenyl)prop-2-enoyl]-1,5-diphenyl-1*H*-pyrazole-4-carbonitrile (**11b**)

Pale yellow powder, 60% yield; mp 209–211 °C; IR (KBr) ν_max_/cm^−1^ 2235 (C≡N); 1667 (C=O), 1595 (C=N); ^1^H NMR (DMSO-*d_6_*) δ 3.82 (s, 3H, -OCH_3_), 7.02 (d, 2H, *J* = 8.8 Hz, ArH), 7.41–7.52 (m, 11H, ArH), 7.6–7.95 (m, 3H, ArH); ^13^C NMR *δ* 56.01 (-OCH_3_), 93.62, 113.83, 115.18, 119.04, 126.65, 129.52, 129.93, 130.00, 130.18, 131.62, 138.54, 145.52, 151.17, 151.65, 162.42, 182.19 (-C=O); MS *m/z* (*%*) 406 (M^+^+1, 0.22), 272 (74.44), 207 (100), 73 (36.91). Anal. Calcd for C_26_H_19_N_3_O_2_ (405.45): C, 77.02; H, 4.72; N, 10.36. Found: C, 77.17; H, 4.67; N, 10.28.

#### 1-(3-Methylphenyl)-5-phenyl-3-[(2*E*)-3-phenylprop-2-enoyl]-1*H*-pyrazole-4-carbonitrile (**11c**)

Pale yellow powder, 69% yield; mp 181–183 °C; IR (KBr) ν_max_/cm^−1^ 2234 (C≡N); 1663 (C=O), 1602 (C=N); ^1^H NMR (DMSO-*d_6_*) δ 2.32 (s, 3H, *m*-CH_3_), 7.16–7.53 (m, 13H, ArH), 7.80–7.98 (m, 3H, ArH); ^13^C NMR *δ* 21.32 (*m*-CH_3_), 93.62, 113.75, 121.63, 123.71, 126.41, 127.00, 129.11, 129.50, 129.58, 129.69, 129.97, 130.84, 131.80, 134.66, 138.47, 139.78, 145.49, 151.21, 151.36, 182.37 (-C=O); MS *m/z* (*%*) 389 (M^+^, 5.28), 207 (100). Anal. Calcd for C_26_H_19_N_3_O (389.45): C, 80.18; H, 4.92; N, 10.79. Found: C, 80.31; H, 5.08; N, 10.74.

#### 3-[(2*E*)-3-(4-Methoxyphenyl)prop-2-enoyl]-1-(3-methylphenyl)-5-phenyl-1*H*-pyrazole-4-carbonitrile (**11d**)

Pale yellow powder, 58% yield; mp 170–172 °C; IR (KBr) ν_max_/cm^−1^ 2234 (C≡N); 1667 (C=O), 1598 (C=N); ^1^H NMR (DMSO-*d_6_*) δ 2.32 (s, 3H, *m*-CH_3_), 3.83 (s, 3H, -OCH_3_), 7.01–7.94 (m, 15H, ArH); ^13^C NMR *δ* 21.32 (*m*-CH_3_), 56.01 (-OCH_3_), 93.55, 113.84, 115.18, 123.71, 126.47, 127.01, 127.32, 129.50, 129.59, 129.96, 130.80, 131.10, 131.62, 138.49, 139.76, 145.48, 151.11, 151.60, 162.43, 182.18 (-C=O); MS *m/z* (*%*) 419 (M^+^, 6.37), 207 (100). Anal. Calcd for C_27_H_21_N_3_O_2_ (419.47): C, 77.31; H, 5.05; N, 10.02. Found: C, 77.22; H, 4.93; N, 9.95.

#### 1-(4-Methylphenyl)-5-phenyl-3-[(2*E*)-3-phenylprop-2-enoyl]-1*H*-pyrazole-4-carbonitrile (**11e**)

Pale yellow powder, 64% yield; mp 262–264 °C; IR (KBr) ν_max_/cm^−1^ 2236 (C≡N); 1668 (C=O), 1597 (C=N); ^1^H NMR (DMSO-*d_6_*) δ 2.35 (s, 3H, *p*-CH_3_), 7.28–7.51 (m, 12H, ArH), 7.79–7.98 (m, 4H, ArH); ^13^C NMR *δ* 21.25 (*p*-CH_3_), 93.60, 113.83, 115.46, 121.58 124.65, 126.41, 127.00, 129.53, 129.69, 129.93, 129.99, 130.33, 131.90, 131.80, 134.12, 139.84, 146.50, 147.69, 151.20, 182.20 (-C=O); MS *m/z* (*%*) 389 (M^+^, 9.65), 207 (200). Anal. Calcd for C_26_H_19_N_3_O (389.45): C, 80.18; H, 4.92; N, 10.79. Found: C, 80.34; H, 4.98; N, 10.85.

#### 3-[(2*E*)-3-(4-Methoxyphenyl)prop-2-enoyl]-1-(4-methylphenyl)-5-phenyl-1*H*-pyrazole-4-carbonitrile (**11f**) [38, 39]

Pale yellow crystals, 66% yield; mp 215–217 °C; IR (KBr) ν_max_/cm^−1^ 2237 (C≡N); 1662 (C=O), 1589 (C=N); ^1^H NMR (DMSO-*d_6_*) δ (s, 3H, *p*-CH_3_), 3.83 (s, 3H, -OCH_3_), 7,03 (d, 2H, J = 9.16 Hz, ArH), 7.28–7.50 (m, 9H, ArH), 7.65–7.95 (m, 4H, ArH); ^13^C NMR *δ* 21.25 (*p*-CH_3_), 56.02 (-OCH_3_), 93.60, 113.84, 115.18, 119.13, 126.41, 126.52, 127.33, 129.51, 129.98, 130.31, 131.60, 136.16, 139.93, 145.48, 151.09, 162.42, 182.21 (-C=O); MS *m/z* (*%*) 419 (M^+^, 4.24), 207 (100). Anal. Calcd for C_27_H_21_N_3_O_2_ (419.47): C, 77.31; H, 5.05; N, 10.02. Found: C, 77.40; H, 4.97; N, 10.16.

### Pharmacology

#### Anti-inflammatory Activity

Adult albino rats of both sexes weighing 120–150 g were obtained from animal house laboratory of Nile Company, Cairo, Egypt and acclimatized for 1 week in the animal facility that has 12 h light/dark cycles with the temperature controlled at 21–23 °C. Normal rat chow and water were made available. The tested compounds and the reference standards were completely dissolved in DMSO. The administered oral dose of the tested compounds was 10 mg/kg body weight with analogy of a reported procedure. Ninety rats were divided into 15 groups each of six animals. All rats were deprived from food and water for 18 h before the experiment and were injected orally by 5 mL water to avoid fluid variation during the process of edema. Two groups received the reference standards; 12 groups received the tested compounds dissolved in DMSO and one group left as negative control group which given 0.2 mL DMSO by oral tube. Indomethacin was obtained from Nile Company for Pharmaceuticals and Chemical Industries, Cairo, Egypt and meloxicam was obtained from Memphis Company for Pharmaceuticals and Chemical Industries, Cairo, Egypt. The tested compounds and meloxicam were given by oral route at doses of 10 mg/kg body weight while indomethacin was given by oral rout at 5 mg/kg body weight. This dose of indomethacin was considered as a positive control for experiments with any new chemical entity [40]. Then, sublunary of 0.1 mL of 2% carrageenan sodium (Sigma, USA) was injected in the right hind paw. The volume of the paw was measured immediately after injection and after administration of the compounds at time intervals 3 and 6 h by using Dial micrometer model (120–1206) Baty, Sussex, England). The results were expressed as volume of edema at each time interval, percentage inhibition of edema volume at each time with respect to control and potency which was calculated compared to indomethacin.

#### Ulcerogenic effects

Ulcerogenic activity of meloxicam, indomethacin and tested compounds were studied in Albino rats of wistar strain weighing 150–200 g of either sex and divided into seven groups each of six animals. The first group served as control group treated with 0.2 mL DMSO. Indomethacin was given by a dose of 5 mg/kg/day while the dose of meloxicam and other tested compounds was 10 mg/kg/day. All groups were treated for three consecutive days by oral tube. During three days of dosage treatment, the animals were starved for 18 h but water was provided *ad libitum*. Food was allowed 2 hours post administration of the drugs. Two hours following the last doses, rats were sacrificed. The stomach of each rat were removed, opened along the greater curvature, rinsed with 0.9% sodium chloride (isotonic solution) and stretched by pins on a cork board. The lesions in gastric mucosa were determined by using stereoscopic microscope. Hemorrhagic lesions were evaluated by scores: 0.0, Normal (no injury, bleeding and latent injury); 0.5, Latent injury or widespread bleeding; 1.0, Slight injury (2 to 3 dotted lines); 2.0, Severe injury (5–6 dotted injuries); 3.0, Very severe injury (several continuous lined injuries) and 4.0, Widespread lined injury or widened injury [33].

### Statistics

In anti-inflammatory study, data are expressed as value ± SEM. Results of carrageenan-induced paw edema experiments are also expressed as percentage of change from control (pre-drug) values. Differences between vehicle control and treatment groups were tested using one-way ANOVA followed by multiple comparisons by the Bonferroni’s test. In ulcerogenic study, Data are presented as mean ± SD and were subjected to one way ANOVA, followed by multiple comparisons by the Bonferroni’s test.

## Figures and Tables

**Sch. 1.**
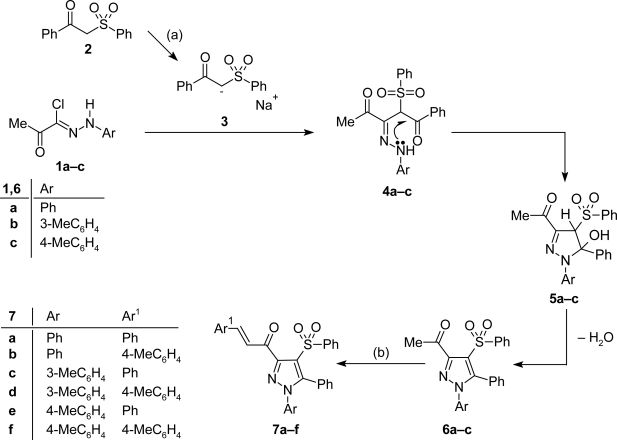
Reagents and conditions: (a) EtONa/EtOH, stirring 12 h r.t.; (b) Ar_1_-CHO, EtOH, 10% NaOH, stirring 12 h, r.t.

**Sch. 2.**
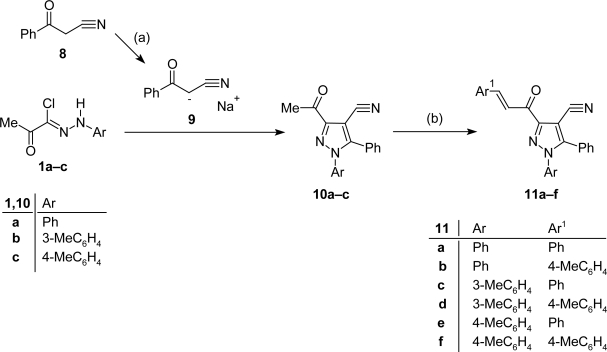
Reagents and conditions: (a) EtONa/EtOH, stirring 12 h, r.t.; (b) Ar_1_-CHO, EtOH, 10% NaOH, stirring 12 h, r.t.

**Tab. 1.** Inhibitory effect of indomethacin, meloxicam and the tested compounds on carrageenan-induced edema of the hind paw in rats

**Group**	**Before injection Edema vol.**	**3 h**			**6 h**		

		**Edema vol.**	**% Edema inhibition**	**Pot. (%)**	**Edema vol.**	**% Edema inhibition**	**Pot. (%)**
Control	0.289 ± 0.018	0.475 ± 0.012	–	0	0.475 ± 0.012	–	0
**6a**	0.243 ± 0.014	0.370 ± 0.017^b^	31.39	68.53	0.378 ± 0.017	27.53	56.39
**6b**	0.244 ± 0.017	0.340 ± 0.018^c^	48.33	105.51	0.341 ± 0.021^b^	48.74	99.82
**6c**	0.256 ± 0.011	0.393 ± 0.018	13.80	30.12	0.356 ± 0.026^a^	21.23	43.48
**7a**	0.248 ± 0.010	0.437 ± 0.015	0.00	0.00	0.447 ± 0.014	0.00	0.00
**7b**	0.249 ± 0.009	0.363 ± 0.020^a^	38.38	83.77	0.366 ± 0.021^a^	36.54	74.83
**7c**	0.290 ± 0.015	0.481 ± 0.011	0.00	0.00	0.491 ± 0.008	0.00	0.00
**7d**	0.257 ± 0.009	0.384 ± 0.011^a^	32.05	69.96	0.368 ± 0.011^a^	40.59	83.12
**7e**	0.259 ± 0.012	0.448 ± 0.006	0.00	0.00	0.457 ± 0.021	0.00	0.00
**7f**	0.260 ± 0.010	0.452 ± 0.007	0.00	0.00	0.458 ± 0.014	0.00	0.00
**10a**	0.256 ± 0.013	0.379 ± 0.021^a^	33.31	72.70	0.365 ± 0.017^a^	41.00	83.97
**10b**	0.241 ± 0.018	0.439 ± 0.005	0.00	0.00	0.443 ± 0.009	0.00	0.00
**10c**	0.251 ± 0.010	0.447 ± 0.009	0.00	0.00	0.438 ± 0.004	0.00	0.00
**11a**	0.248 ± 0.009	0.439 ± 0.011	0.00	0.00	0.446 ± 0.006	0.00	0.00
**11b**	0.239 ± 0.009	0.391 ± 0.013	17.88	39.04	0.386 ± 0.045	20.50	41.99
**11c**	0.252 ± 0.010	0.422 ± 0.019	8.08	17.64	0.414 ± 0.019	12.59	25.78
**11d**	0.261 ± 0.009	0.451 ± 0.018	0.00	0.00	0.438 ± 0.020	4.94	10.11
11**e**	0.253 ± 0.016	0.419 ± 0.016	10.85	23.69	0.410 ± 0.014	15.54	31.83
**11f**	0.262 ± 0.011	0.424 ± 0.007	12.52	27.34	0.419 ± 0.011	15.36	31.46
Meloxicam	0.279 ± 0.009	0.385 ± 0.017^a^	42.65	93.10	0.375 ± 0.018	48.25	98.82
Indomethacin	0.263 ± 0.016	0.364 ± 0.011^b^	45.81	100	0.358 ± 0.017^a^	48.83	100

Values represent means ± SEM of six animals for each group. The potency (pot.) was calculated comparedto the reference drug indomethacin. Statistical analysis using One-way ANOVA (Bonferroni’s multiple comparison test). Significance levels

a *P < 0.05*;

b *P< 0.01*and

c *P < 0.001*as compared with control.

**Tab. 2.** Ulcer index of indomethacin, meloxicam, **6b, 7b, 7d and 10a.**

**Compound**	**Ulcer index^a^**
Control	0.25 ± 0.274
**6b**	1.5±1.000
**7b**	2 ± 0.894^b^
**7d**	1.83 ± 0.983
**10a**	2.83 ± 0.753^c^
Meloxicam	1.67 ± 0.817
Indomethacin	3.17 ± 0.753^c^

a Values represent means ± SD (n = 6);

Significance level b, *P* < 0.05;

c, P < 0.01 as compared with the respective control.

**Tab. 3.** Docking results of SC-558 and the tested compounds with active site of COX-2.

**Cpd.**	**Conformational stack energy (kcal/mol)**	**H-bonds**	**Amino acid residue(s) of H-bond(s) (bond length Å)**
SC-558	–88.3709	5	Arg513 (2.20), His90 (2.30), Gln192 (2.79), Ser353 (1.51) & Tyr355 (2.16)
**6a**	−63.6787	5	Tyr355 (1.03), His90 (2.51, 2.62) & Arg513 (1.76, 2.58)
**6b**	−67.6756	5	Tyr355 (1.09), His90 (2.39, 2.50) & Arg513 (1.82, 2.73)
**6c**	−54.3336	5	Tyr355 (1.07), His90 (2.45, 2.48) & Arg513 (1.62, 2.55)
**7b**	−57.7361	3	Tyr355 (0.95), His90 (2.66) & Arg513 (2.45)
**7d**	−64.2746	3	Tyr355 (1.01), His90 (2.59) & Arg513 (2.09)
**10a**	−57.7051	2	Arg120 (2.61) & Tyr355 (2.21)
**11b**	−52.7570	2	Arg120 (2.55) & Tyr355 (2.10)
**11c**	−43.5693	3	Arg513 (2.24) & Tyr355 (1.44)
**11e**	−49.9558	2	His90 (2.51) & Tyr355 (2.01)
**11f**	−44.2973	3	Arg513 (2.36) & Tyr355 (1.63)
